# Associations Between Metabolic Risk Factors and Lung Function Among Adults in Northern Thailand: A Cross-Sectional Study

**DOI:** 10.3390/healthcare13141671

**Published:** 2025-07-10

**Authors:** Anurak Wongta, Nan Ei Moh Moh Kyi, Muhammad Samar, Nyan Lin Thu, Tipsuda Pintakham, Surat Hongsibsong

**Affiliations:** 1 School of Health Sciences Research, Research Institute for Health Sciences, Chiang Mai University, Chiang Mai 50200, Thailand; naneimohmoh_kyi@cmu.ac.th (N.E.M.M.K.); muhammad_samar@cmu.ac.th (M.S.); nyanlinthu_1@cmu.ac.th (N.L.T.); tipsuda_pintakham@cmu.ac.th (T.P.); 2 Environmental, Occupational, and NCD Center of Excellent, Research Institute for Health Sciences, Chiang Mai University, Chiang Mai 50200, Thailand

**Keywords:** lung function, metabolic risk factors, 6-min walk test, respiratory impairment, Thailand population health

## Abstract

Background/Objectives: Lung function decline is influenced by metabolic risk factors (e.g., obesity, hyperglycemia, dyslipidemia) and environmental exposures (e.g., PM2.5), which may jointly contribute to airway inflammation and lung function impairment. This study aimed to investigate these associations in northern Thai adults and identify factors linked to lung function impairment. Methods: A cross-sectional study was conducted in San Pa Thong, Chiang Mai, Thailand, involving 137 adults. Data on metabolic indicators and spirometry were collected. Statistical analyses included Spearman’s correlation, multivariable linear regression, and logistic regression. Results: Higher triglyceride levels and shorter 6-min walk test (6MWT) distances were associated with reduced forced expiratory volume in one second (FEV_1_) and forced vital capacity (FVC). Only 6MWT distance remained a significant factor for lung function impairment in logistic regression (adjusted OR = 0.763, 95% CI: 0.588–0.990, *p* = 0.042). Conclusions: Combining metabolic and respiratory assessments may improve early detection of lung function impairment in high-risk populations, particularly given the dual burden of metabolic disorders and air pollution in northern Thailand. These findings support the integration of metabolic and respiratory screening in community health programs to enhance preventive strategies.

## 1. Introduction

Lung function is a critical indicator of respiratory health, as it affects overall morbidity and mortality. Various health issues, such as respiratory and cardiovascular diseases, cognitive decline, metabolic syndrome, and elevated mortality risks, are correlated with diminished lung function [1,2]. A primary tool known as spirometry is used for evaluating pulmonary function and identifying respiratory dysfunction patterns. This technique measures essential parameters, including forced expiratory volume in one second (FEV_1_), forced vital capacity (FVC), and determines their ratio (FEV_1_/FVC) [3].

Growing evidence suggests that metabolic health significantly influences lung function. Obesity is associated with restrictive and obstructive ventilatory patterns, which are likely the result of systemic inflammation and mechanical constraints [4,5]. A decline in lung volumes associated with elevated blood glucose and HbA1c levels might result from chronic inflammation and microvascular changes [6]. While going through a variety of population studies, dyslipidemia, particularly elevated triglyceride levels, has been associated with impaired lung function, even among those individuals who do not have a formal diagnosis of pulmonary disease [7,8]. Such types of metabolic factors may contribute to early respiratory decline, even in those without diagnosed pulmonary disease [6]. Agricultural burning, vehicular emissions, and wildfires, which are prevalent in southern and northern Thailand, pose public health threats by elevating the level of PM2.5 and air pollution. An exposure to PM2.5 is associated with inflammation, airway remodelling, and a decline in lung function, supported by a growing body of evidence [9,10,11,12]. Environmental variables might aggravate the negative consequences of metabolic diseases on lung function. In Thailand, rapid urbanization, high rates of metabolic syndrome, and seasonal agricultural burning uniquely converge to exacerbate lung function decline compared to other settings. Emerging evidence indicates that environmental pollutants, particularly PM2.5, may induce systemic inflammation, contributing to metabolic disorders that potentially affect lung function.

Importantly, metabolic risk factors (such as obesity, diabetes, and dyslipidemia) exacerbate lung impairment through systemic inflammation, oxidative stress, and microvascular damage [13,14]. Environmental exposure, particularly to PM2.5 and industrial pollutants, further worsens respiratory health by inducing airway inflammation and remodeling [15,16]. Their synergistic impact is especially concerning in Thailand due to high pollution levels and a growing prevalence of metabolic disorders [17,18,19]. Integrating these risk factors into health screening may aid in early detection and prevention strategies for lung impairment.

Integrating metabolic and respiratory evaluations in community health screening is essential to address these dual health risks. This approach is particularly relevant in Southeast Asia, where metabolic and respiratory disorders cause major problems for healthcare systems. Community-driven screening initiatives facilitate early detection and preventive measures in resource-constrained environments.

Across many cultures, the correlation between metabolic disorders and pulmonary function has been investigated; most of the research has occurred in Western nations [1,7]. There exists a lack of data from Southeast Asia, particularly Thailand, where lung health may be impacted in a manner that is distinct from other regions due to the increasing prevalence of metabolic disorders and changing lifestyles. Additionally, multiple prior investigations have concentrated exclusively on obstructive lung patterns or FEV_1_/FVC ratios, neglecting the analysis of associated factors in both continuous and categorical clinical forms or restrictive impairment. It is crucial to address these gaps to gain a more comprehensive understanding of population-specific risk factors and to inform targeted prevention strategies [20,21,22].

The study aimed to explore the connection between metabolic risk factors and lung function in Thai adults. The specific aims included: (1) analyzing the relationships between metabolic risk factors and lung function metrics; and (2) determining factors linked to lung function decline through multivariable analyses accounting for age and gender. The findings from this study may inform community health interventions, such as integrated metabolic and respiratory health screenings, and support policy reforms aimed at reducing respiratory disease burdens in regions with overlapping metabolic and environmental risks.

## 2. Materials and Methods

### 2.1. Study Design and Participants

This study was conducted in San Pa Thong District, Chiang Mai Province, Thailand. A cross-sectional design was employed to effectively assess the relationship between metabolic risk factors and lung function at one point in time, suitable for extensive health surveys [23]. Nonetheless, it has limitations, including the inability to determine causality and the risk of selection bias. One hundred fifty-four participants were recruited from the local community through health awareness events and screening programs that took place from 30 October to 30 December 2023. Of these, 137 participants produced acceptable and reproducible spirometry results and were included in the final analysis. Inclusion criteria included adults aged ≥18 years who did not have walking disabilities or were not bedridden. Exclusion criteria included pregnancy, known chronic respiratory diseases (e.g., COPD, asthma), or inability to perform spirometry. This ensured generalizability while maintaining participant safety.

### 2.2. Data Collection Procedures

Data collection was conducted through structured interviews, physical assessments, laboratory testing, and pulmonary function tests. Demographic data, smoking status, and medical history were documented by the trained person on the team.

Anthropometric parameters included height, weight, and body mass index (BMI), derived from weight in kilograms (kg) divided by height in meters squared (m^2^). Blood pressure, heart rate, and peripheral oxygen saturation (SpO_2_) were checked before and right after the six-minute walk test (6MWT), following the standard rules. Functional exercise capacity was evaluated by measuring the total distance achieved during the six-minute walking protocol.

Blood samples were taken in the morning, after participants had fasted overnight. Samples were used to measure fasting blood sugar, HbA1c, cholesterol, and triglyceride levels. All laboratory tests and assessments were performed using standard methods at the Prompt Health Centre, Faculty of Associated Medical Sciences, Chiang Mai University.

Following the earlier studies, lung health assessment was performed using a SpiroScout spirometer (Ganshorn, UK), recording FEV_1_, FVC, and FEV_1_/FVC [24]. We conducted spirometry by adhering to the American Thoracic Society/European Respiratory Society (ATS/ERS) recommendations [25]. Each participant completed at least three acceptable and repeat maneuvers; the best values were used for analysis.

Spirometry is a reliable and widely accessible tool for assessing lung function; however, it may underestimate subclinical disease compared to advanced methods such as high-resolution computed tomography (HRCT) [26,27] and diffusing capacity for carbon monoxide (DLCO) [28], which can detect early parenchymal and airway involvement. Similarly, the 6MWT is a low-cost functional assessment, but results may vary with participant effort. While advanced methods may be more sensitive, they are not feasible in large community-based studies due to cost and logistics.

### 2.3. Definition of Lung Function Impairment

Lung function was categorized into two possibilities: either normal or impaired. Impaired lung function was characterized by an obstructive (FEV_1_/FVC < 70%) or restrictive pattern (FEV_1_/FVC ≥ 70% with FVC < 80% of predicted). These definitions adhered to international spirometry standards, considering age, sex, and height.

### 2.4. Statistical Analysis

Statistical analyses were carried out using the IBM SPSS Statistics program, version 20. Continuous variables were reported as median with interquartile range (IQR), and categorical variables as frequencies and percentages. Continuous data were evaluated by the Mann–Whitney U test, and categorical data by Fisher’s exact test for differences between normal and impaired lung function groups. Spearman’s rank correlation coefficient was used to assess correlations between continuous metabolic-associated factors and lung function parameters. Multivariable linear regression models were used to analyze relationships between metabolic-associated factors (continuous and dichotomized clinical cutoffs) and lung function parameters (FEV_1_, FVC, and FEV_1_/FVC). Associated factors of lung function impairment were assessed using binary logistic regression, adjusting for age and gender. Statistical significance was defined as a *p*-value < 0.05.

### 2.5. Ethical Approval and Consent

All participants provided written informed consent prior to data collection. Ethical approval was granted by the Faculty of Associated Medical Technology, Chiang Mai University (Approval No. AMSEC-66EX-062), approval date 1 April 2020. Participant confidentiality was maintained using coded identifiers, secure data storage, and anonymization prior to analysis. Data collectors were trained in privacy protocols to protect participants’ information during spirometry and metabolic health data collection. Any ethical challenges encountered (e.g., participant anxiety regarding spirometry) were mitigated through pre-test counseling and informed consent procedures.

## 3. Results

### 3.1. Participant Characteristics

The study included 137 subjects overall; 118 (86.1%) were classified as having a normal lung function, and 19 (13.9%) as having a reduced lung function. Table 1 shows the baseline characteristics categorized by lung function status. The median age was 62 years for those with impaired lung function and 60 years for those with normal function, indicating that individuals with impaired lung function were notably older (*p* = 0.024). Regarding smoking status or sex distribution, there were no significant variations between the groups. 

Among metabolic factors, those with reduced lung function had notably higher fasting blood sugar levels (median 120 vs. 96 mg/dL, *p* = 0.002) and higher HbA1c values (median 6.5% vs. 5.9%, *p* = 0.006). In the impaired group (52.6% vs. 29.7%, *p* = 0.048), the proportion of patients with HbA1c ≥ 6.5% was also significantly higher. Though the impaired group had generally lower cholesterol levels, the difference was not statistically significant (*p* = 0.059). BMIs, triglyceride levels, or the proportion of participants with BMI ≥25 kg/m^2^ or triglycerides ≥150 mg/dL showed no notable differences between the groups.

Participants with impaired lung function exhibited lower spirometry measures, specifically FEV_1_ (1.4 vs. 1.9 L, *p* < 0.001) and FVC (1.7 vs. 2.2 L, *p* = 0.001). No significant difference was noted in the FEV_1_/FVC ratio between the groups (*p* = 0.325). Furthermore, the 6-min walk test (6MWT) revealed a significantly reduced distance in the impaired group (389 vs. 440 m, *p* = 0.004), demonstrating a decline in functional capacity.

### 3.2. Correlations Between Metabolism-Associated Factors and Lung Function

Spearman’s correlation analysis revealed several significant associations between metabolic and functional variables and lung function parameters (Table 2). Age was negatively correlated with both FEV_1_ (ρ = −0.369) and FVC (ρ = −0.332, *p* < 0.01), indicating that lung function declines with advancing age. HbA1c was also negatively associated with FEV_1_ (ρ = −0.231, *p* < 0.01) and positively correlated with the FEV_1_/FVC ratio (ρ = 0.183, *p* < 0.05), suggesting a complex relationship between glycemic control and lung function.

Triglycerides were negatively correlated with FEV_1_ (ρ = −0.177, *p* < 0.05), with no significant correlations to FVC or the FEV_1_/FVC ratio. BMI and fasting blood sugar lacked significant correlations with spirometry outcomes.

Importantly, functional capacity as measured by 6MWT distance was positively associated with both FEV_1_ (ρ = 0.168, *p* < 0.05) and FVC (ρ = 0.219, *p* < 0.01), indicating that greater physical performance is linked to better lung function. No significant correlation was found between 6MWT distance and the FEV_1_/FVC ratio. 

### 3.3. Multivariable Linear Regression: Continuous Associated Factors

Table 3 provides the multivariable linear regression model evaluations of determinants of lung function metrics. Being male was connected to increased FEV_1_ (B = 0.880, 95% CI: 0.710 to 1.040, *p* < 0.001) and FVC (B = 1.110, 95% CI: 0.940 to 1.290, *p* < 0.001). Older age was associated with reduced FEV_1_ and FVC; however, no significant association was seen for the FEV_1_/FVC ratio.

Lower FEV_1_ and FVC values associated with higher triglyceride levels suggest that abnormal fat levels in the blood may contribute to reduced lung capacities (FEV_1_: B = −0.001, 95% CI: −0.002 to 0.000, *p* = 0.050; FVC: B = −0.002, 95% CI: −0.003 to −0.001, *p* = 0.001). In the adjusted models, HbA1c, fasting glucose, BMI, and cholesterol showed no significant association with any of the three spirometry outcomes.

In the continuous model, the distance computed by the 6MWT had no substantial effect on FEV_1_, FVC, or the FEV_1_/FVC ratio. After adjustment, smoking status also displayed no significant correlations with any measures of pulmonary function. 

### 3.4. Multivariable Linear Regression: Categorical Associated Factors

To improve clinical interpretability, a secondary regression analysis was performed using associated factors categorized according to standard clinical cutoffs. Table 4 presents the multivariable linear regression results for FEV_1_, FVC, and the FEV_1_/FVC ratio using these categorized variables. 

Men exhibited greater lung volumes than women, with increases of 0.841 L in FEV_1_ and 1.053 L in FVC (*p* < 0.001). However, gender was not associated with differences in the FEV_1_/FVC ratio. Similarly, individuals aged 60 and older had significantly lower FEV_1_ and FVC values compared to younger participants (*p* < 0.001), though age had no apparent effect on the FEV_1_/FVC ratio. 

Among the metabolic risk factors, having triglyceride levels of 150 mg/dL or higher was linked to significantly lower FEV_1_ and FVC values (*p* < 0.001). In contrast, BMI ≥25 kg/m^2^ and HbA1c ≥6.5% showed no meaningful association with any of the lung function measures. Similarly, smoking status was not significantly related to FEV_1_, FVC, or the FEV_1_/FVC ratio in the adjusted models.

### 3.5. Partially Adjusted Logistic Regression Predicting Lung Function Impairment

We used logistic regression to explore how categorized metabolic and functional factors relate to lung function impairment (Table 5). Participants with HbA1c levels of 6.5% or higher had a higher likelihood of lung impairment, but this trend was not statistically significant (*p* = 0.053). Similarly, elevated triglycerides (≥150 mg/dL) and BMI ≥25 kg/m^2^ were not significantly associated with impairment in either crude or adjusted models.

In contrast, functional performance measured by the 6MWT was strongly associated with lung health. For every 50-m increase in walking distance, the odds of having an impaired lung function dropped by about 28% at *p* = 0.020. This relationship remained significant even after adjusting for age and gender, underscoring the importance of physical capacity as an independent predictor of respiratory status.

## 4. Discussion

This study explored the connection between metabolic health signs and lung function in Thai adults from northern Thailand. The findings reveal that there are solid associations between age, gender, triglyceride levels, and 6-min walk test distance with key spirometry results, mainly FEV_1_ and FVC. Notably, the 6MWT distance stood out as the sole factor independently tied to impaired lung function in the adjusted regression model. On the other end, HbA1c levels ≥6.5% initially appeared to increase risk in unadjusted analyses. This association lost significance once other variables were accounted for.

Our results are similar to earlier studies and show that lung function usually becomes worse as people become older, probably due to less flexible chest walls, weaker breathing muscles, and narrower airways [29,30]. As anticipated, male participants exhibited higher FEV_1_ and FVC measurements compared to females, differences reflecting established physiological disparities in lung capacity and airway structure between sexes [31]. Results also showed that when triglyceride levels reach 150 mg/dL or above, they were strongly associated with reduced lung volumes across both continuous and grouped analyses. This supports earlier studies indicating that imbalances in blood lipids, dyslipidemia, could harm lung function by increasing chronic inflammation or disrupting blood vessel health [32,33,34]. This study deepens our understanding of how metabolic risk factors (particularly triglycerides) interact with lung function decline in a Southeast Asian context. It highlights the potential for systemic inflammation and vascular changes to drive early lung function deterioration, even in community-based populations.

Interestingly, although HbA1c and BMI showed weak correlations with lung function in unadjusted analyses, they did not remain significant associated factors after controlling for other factors. This may be due to collinearity with other metabolic variables or the limited statistical power of the sample size, aligning with studies that reported inconsistent associations between glucose metabolism and lung function [35,36,37]. This contrasts with some earlier studies that reported stronger links between the elevated blood sugar levels and reduced lung function [6,38,39], or between prolonged obesity and breathing difficulties [5,40]. This highlights the complexity of assessing the independent contributions of metabolic factors to lung function in different populations. Our findings reinforce the known relationship between obesity and restrictive lung patterns, likely due to mechanical constraints and low-grade inflammation.

Notably, while the 6MWT distance was a significant predictor in logistic regression for lung function impairment, it was not consistently significant in linear models. This suggests that the 6MWT may capture aspects of functional capacity relevant to categorical impairment but not always to continuous spirometry values. This distinction underscores the importance of interpreting functional tests like the 6MWT carefully, depending on the statistical approach and outcome measure.

The 6MWT distance emerged as the most consistent predictor across all analytic models. It was positively correlated with FEV_1_ and FVC in correlation and linear regression and independently associated with reduced odds of lung impairment. This emphasizes the 6MWT’s value as a practical, low-cost screening tool for respiratory risk, particularly in resource-limited settings [41,42,43,44]. Additional studies have shown strong correlations between 6MWT distance and lung function measures like FEV_1_/FVC ratio, affirming its validity for subclinical detection [45,46,47].

This research adds to existing work exploring how metabolic health and lung function interact, with a focus on Thailand. In northern regions like Chiang Mai, seasonal air quality issues—such as smoke from agricultural burning and regional haze—may worsen breathing challenges [9,17,48,49]. During dry months, Chiang Mai’s PM2.5 levels often rise far above global safety standards, which could accelerate subtle lung damage in people already at metabolic risk [50,51,52]. These environmental factors may interact synergistically with metabolic disorders, highlighting the need for integrated community health interventions to mitigate their combined impact.

From a public health perspective, the findings highlight the value of combining screenings for chronic diseases that look at both metabolic and respiratory health. Tools like the 6MWT and routine lipid screening could aid in early identification and intervention to preserve lung health [53]. Such strategies may be feasible through Thailand’s existing non-communicable disease (NCD) prevention infrastructure, enhancing early detection and risk stratification [54].

### 4.1. Strengths and Limitations

This study’s strengths include community-based recruitment, use of standardized spirometry, and comprehensive assessment of metabolic and functional variables. Limitations include the cross-sectional design, which precludes causal inference, potential residual confounding from unmeasured variables, and a relatively small sample size.

### 4.2. Implications of Findings

Integrating metabolic and respiratory screening in community health settings could enable earlier detection of lung function decline. These findings support incorporating the 6MWT and triglyceride screening into Thailand’s NCD programs and educating communities about PM2.5 exposure reduction to prevent further lung function deterioration. Policymakers in Thailand should consider adding 6MWT and lipid screening to routine check-ups, particularly in areas with high PM2.5 exposure. Globally, the findings highlight the importance of integrating metabolic and respiratory evaluations in community health programs, especially in regions facing high burdens of both metabolic disorders and air pollution.

## 5. Conclusions

This study demonstrates a significant association between elevated triglyceride levels and reduced lung function among Thai adults. The findings establish triglycerides as a key metabolic predictor of respiratory health decline in this Southeast Asian population. While the 6-min walk test showed variable significance across models, metabolic markers consistently predicted lung function outcomes. These results provide new evidence for the metabolic-respiratory health connection in Thai adults and support the integration of lipid screening in respiratory health assessments.

## Figures and Tables

**Table 1:** Baseline characteristics of participants by lung function status.

Variable	Total (N = 137)	Lung Function Impairment		*p*-Value
		Normal (*n* = 118)	Impaired (*n* = 19)	
Demographic characteristics				
Age, years	60 (55–65)	60 (54–65)	62 (60–67)	0.024 *
Male (Male)	30 (21.9%)	27 (22.9%)	3 (15.8%)	0.765
Smoking (Yes)	16 (11.7%)	14 (11.9%)	2 (10.5%)	1.000
Anthropometric and metabolic				
BMI, kg/m^2^	24.3 (21.5–25.9)	24.3 (21.5–26.0)	24.9 (20.8–26.5)	0.813
Fasting blood sugar, mg/dL	98 (91–116)	96 (90–111)	120 (101–135)	0.002 *
HbA1c, %	6.1 (5.6–6.6)	5.9 (5.5–6.5)	6.5 (6.1–7.6)	0.006 *
Cholesterol, mg/dL	202 (172–229)	205 (177–231)	181(166–226)	0.059
Triglyceride, mg/dL	116 (84–168)	111 (84–164)	146 (89–185)	0.304
Categorical metabolic markers				
BMI ≥ 25 kg/m^2^	57 (41.6%)	48 (40.7%)	9 (47.4%)	0.583
HbA1c ≥ 6.5%	45 (32.8%)	35 (29.7%)	10 (52.6%)	0.048 *
Triglycerides ≥ 150 mg/dL	47 (34.3%)	38 (32.2%)	9 (47.4%)	0.196
Lung function and capacity				
FEV_1_, L	1.8 (1.6–2.1)	1.9 (1.7–2.2)	1.4 (1.3–1.5)	<0.001 **
FVC, L	2.2 (2.0–2.5)	2.2 (2.0–2.6)	1.7 (1.6–2.2)	0.001 *
FEV_1_/FVC ratio, %	85.3 (78.2–90.3)	85.3 (79.3–90.5)	86.8 (67.1–88.7)	0.325
6MWT, meters	435 (372–484)	440 (384–508)	389 (326–420)	0.004 *

Abbreviations: BMI, body mass index; FBS, fasting blood sugar; HbA1c, glycated hemoglobin; TG, triglyceride; FEV_1_, forced expiratory volume in 1 s; FVC, forced vital capacity; 6MWT, 6-min walk test. * *p*-value < 0.05; ** *p*-value < 0.001.

**Table 2:** Correlation coefficients between metabolic-associated factors and lung function parameters.

Predictor Variable	FEV_1_ (ρ)	FVC (ρ)	FEV_1_/FVC (ρ)
Age (years)	−0.369	−0.332 **	0.085
BMI (kg/m^2^)	−0.012	0.046	0.08
FBS (mg/dL)	−0.107	−0.028	0.104
HbA1C (%)	−0.231 **	−0.119	0.183 *
Cholesterol (mg/dL)	0.100	0.097	0.009
Triglyceride (mg/dL)	−0.177 *	−0.096	0.074
6MWT Distance (m)	0.168 *	0.219 **	0.071

Abbreviations: ρ = Spearman’s correlation coefficient. Correlations were assessed using Spearman’s rank correlation coefficient (ρ). A *p*-value of less than 0.05 was considered statistically significant. Only continuous metabolic-associated factors are included. Abbreviations: FEV_1_, forced expiratory volume in 1 s; FVC, forced vital capacity; TG, triglyceride; HbA1c, glycated hemoglobin; 6MWT, 6-min walk test. * *p*-value < 0.05; ** *p*-value < 0.001.

**Table 3:** Multivariable linear regression predicting lung function using continuous associated factors.

Predictor	FEV_1_		FVC		FEV_1_/FVC Ratio, %	
	B (95% CI)	*p*-Value	B (95% CI)	*p*-Value	B (95% CI)	*p*-Value
Gender (Male)	0.880 (0.710–1.040)	<0.001 **	1.110 (0.940–1.290)	<0.001 **	−1.530 (−5.790–2.720)	0.477
Smoking (Yes)	−0.140 (−0.350–0.080)	0.206	−0.110 (−0.340–0.120)	0.334	−2.620 (−8.120 −2.880)	0.348
Age (years)	−0.020 (−0.029–−0.011)	<0.001 **	−0.023 (−0.033–−0.014)	<0.001 **	0.031 (−0.203–0.264)	0.795
BMI (kg/m^2^)	−0.001 (−0.018–0.016)	0.884	−0.007 (−0.026–0.011)	0.426	0.201 (−0.244–0.645)	0.373
Glucose (mg/dL)	−0.001 (−0.003–0.002)	0.706	0.00007 (−0.003–0.003)	0.962	−0.017 (−0.085–0.052)	0.627
HbA1c (%)	0.001 (−0.065–0.066)	0.986	−0.035 (−0.105–0.035)	0.324	1.190 (−0.510–2.890)	0.168
Cholesterol (mg/dL)	0.001 (−0.001–0.002)	0.224	0.001 (−0.001–0.003)	0.222	0.002 (−0.036–0.040)	0.931
Triglyceride (mg/dL)	−0.001 (−0.002–0.000)	0.050 *	−0.002 (−0.003–−0.001)	0.001 **	0.014 (−0.009–0.038)	0.229
6MWT distance (meters)	0.00001 (0.000–0.000)	0.939	−0.0001 (0.000–0.000)	0.588	0.004 (−0.005–0.013)	0.351

Model Fit: FEV_1_: R = 0.739, R^2^ = 0.546, Adjusted R^2^ = 0.513, FVC: R = 0.796, R^2^ = 0.633, Adjusted R^2^ = 0.607, FEV_1_/FVC: R = 0.259, R^2^ = 0.067, Adjusted R^2^ = 0.01. Values are unstandardized regression coefficients (B) with corresponding 95% confidence intervals (CI). Models were adjusted for age, gender, smoking, BMI, fasting glucose, HbA1c, cholesterol, triglycerides, and 6-min walk distance. A *p*-value of less than 0.05 was considered statistically significant. Abbreviations: FEV_1_, forced expiratory volume in 1 s; FVC, forced vital capacity; TG, triglyceride; HbA1c, glycated hemoglobin; 6MWT, 6-min walk test. * *p*-value < 0.05; ** *p*-value < 0.001.

**Table 4:** Multivariable linear regression predicting lung function parameters using binary clinical cutoffs.

Predictor	FEV_1_ (L)		FVC (L)		FEV_1_/FVC Ratio, %	
	B (95% CI)	*p*-Value	B (95% CI)	*p*-Value	B (95% CI)	*p*-Value
Gender (Male)	0.841 (0.679–1.004)	<0.001 **	1.053 (0.878–1.229)	<0.001 **	−1.037 (−5.293–3.219)	0.631
Smoking (Yes)	−0.153 (−0.361–0.056)	0.150	−0.119 (−0.345–0.107)	0.299	−2.884 (−8.356–2.587)	0.299
Age ≥ 60 years	−0.262 (−0.383–0.142)	<0.001 **	−0.308 (−0.438–0.178)	<0.001 **	0.075 (−3.080–3.230)	0.963
BMI ≥ 25 kg/m^2^	0.048 (0.168–0.071)	0.426	−0.093 (−0.222–0.037)	0.160	1.230 (−1.913–4.373)	0.44
HbA1c ≥ 6.5%	−0.035 (−0.162–0.092)	0.585	−0.082 (−0.220–0.055)	0.239	2.159 (−1.177–5.496)	0.203
Triglyceride ≥ 150 mg/dL	−0.187 (−0.312–0.061)	<0.001 **	−0.249 (−0.384–0.113)	<0.001 **	0.494 (−2.793–3.781)	0.767

Model Fit: FEV_1_: R = 0.734, R^2^ = 0.539, Adjusted R^2^ = 0.517, FVC: R = 0.786, R^2^ = 0.618, Adjusted R^2^ = 0.600, FEV_1_/FVC ratio: R = 0.187, R^2^ = 0.035, Adjusted R^2^ = −0.009 Values are unstandardized regression coefficients (B) with 95% confidence intervals (CI). All models were adjusted for gender, smoking status, and age ≥60 years. Categorical associated factors were coded as 1 for abnormal or elevated and 0 for normal. A *p*-value of less than 0.05 was considered statistically significant. Abbreviations: FEV_1_, forced expiratory volume in 1 s; FVC, forced vital capacity; TG, triglyceride; HbA1c, glycated hemoglobin; 6MWT, 6-min walk test. ** *p*-value < 0.001.

**Table 5:** Partially adjusted logistic regression for lung function impairment.

Predictor	Lung Function Impairment			
	Crude OR (95% CI)	*p*-Value	Adjusted OR (95% CI)	*p*-Value
BMI ≥ 25	1.312 (0.496–3.471)	0.584	1.439 (0.528–3.917)	0.477
HbA1c ≥ 6.5%	2.635 (0.986–7.044)	0.053	2.299 (0.837–6.311)	0.106
TG ≥ 150 mg/dL	1.895 (0.711–5.048)	0.201	1.930 (0.699–5.331)	0.205
6MWT distance (per 50 m)	0.719 (0.544–0.949)	0.020 *	0.763 (0.588–0.990)	0.042 *

Crude and adjusted odds ratios (OR) are presented with 95% confidence intervals. Each adjusted model included gender and age ≥60 years as covariates. Lung function impairment included obstructive and restrictive patterns. A *p*-value of less than 0.05 was considered statistically significant. Abbreviations: OR, odds ratio; CI, confidence interval; TG, triglyceride; HbA1c, glycated hemoglobin; 6MWT, 6-min walk test. * *p*-value < 0.05.

## Data Availability

The original contributions presented in the study are included in the article; further inquiries can be directed to the corresponding author.
